# Development of computer adaptive tests to assess the psychological status of individuals with an eating disorder or type 2 diabetes

**DOI:** 10.1186/s13030-025-00325-z

**Published:** 2025-02-14

**Authors:** Takeshi Horie, Ken Kurisu, Shuji Inada, Kenshi Kawahara, Yutaka Matsuyama, Hiroe Kikuchi, Yoshiharu Yamamoto, Toshimasa Yamauchi, Kazuhiro Yoshiuchi

**Affiliations:** 1 Department of Stress Sciences and Psychosomatic Medicine, Graduate School of Medicine, The University of Tokyo, Tokyo, Japan; 2 Department of Psychosomatic Medicine, Saitama Cancer Center, Saitama, Japan; 3 Nozomi Clinic, Kanagawa, Japan; 4 Department of Biostatistics, School of Public Health, Graduate School of Medicine, The University of Tokyo, Tokyo, Japan; 5 Department of Psychosomatic Medicine, National Center for Global Health and Medicine Hospital, Tokyo, Japan; 6 Educational Physiology Laboratory, Graduate School of Education, The University of Tokyo, Tokyo, Japan; 7 Department of Diabetes and Metabolic Diseases, Graduate School of Medicine, The University of Tokyo, Tokyo, Japan

**Keywords:** Computerized adaptive testing, Item response theory, Eating disorders, Type 2 diabetes, Hospital anxiety and Depression Scale, Eating behaviors

## Abstract

**Background:**

Individuals with type 2 diabetes and eating disorders must change their eating behaviors, which are often influenced by psychological factors like depression and anxiety. To efficiently assess daily psychological status, the present study aimed to develop computerized adaptive tests (CAT) based on item response theory (IRT).

**Methods:**

Individuals with depression, anxiety disorders, eating disorders, type 2 diabetes, and healthy persons participated in the study. Participants completed six questionnaires, including momentary and most recent one-week depression, anxiety, and positive affect. We selected items meeting the IRT assumptions, applied a graded response model, and conducted CAT simulations.

**Results:**

Across all six questionnaires, the CAT simulations used a smaller number of items and exhibited substantial Pearson’s correlation coefficients exceeding 0.95 between simulated and full item-set mood status estimates. These estimated mood scores demonstrated satisfactory concurrent validity with the Hospital Anxiety and Depression Scale and sufficient discriminant validity between the clinical group and healthy controls.

**Conclusion:**

These findings suggest that these scales offer efficient measurement of the mood status of individuals with an eating disorder or type 2 diabetes.

**Supplementary Information:**

The online version contains supplementary material available at 10.1186/s13030-025-00325-z.

## Introduction

The increase in the prevalence of type 2 diabetes represents a public health concern, with lifestyle modifications like regular diet and exercise habits being crucial for glycemic management [1]. The eating behaviors of people with type 2 diabetes are influenced by psychological factors, including stress and lack of sleep [2, 3]. Eating disorders are also highly prevalent, especially among women [4], with a reported increase in prevalence after the COVID-19 pandemic [5]. Psychological factors, such as depression and anxiety, can cause uncontrollable eating behavior [6–8]. Self-monitoring of both physical status and the psychological aspects of daily life is essential [9, 10], and daily mood assessment is important for individuals with type 2 diabetes or eating disorders.

Self-administered questionnaires are widely used to measure mood status, such as depression and anxiety [11, 12]. These scales were developed based on classical test theory, which has several limitations. These include the inability to compare different scales due to item dependency, the difficulty in comparing different groups of examinees due to sample dependency, the requirement to answer all items regardless of severity for scoring, and restrictions against replacing or adding items. Computer adaptive testing (CAT), based on item response theory (IRT), can overcome these limitations. CAT adjusts the item presentation based on the subject’s responses, allowing for highly accurate measurement with fewer items [13, 14].

The U.S. National Institutes of Health developed the Patient-Reported Outcomes Measurement Information System (PROMIS) project to assess patient-reported outcomes, including emotional distress [15]. The PROMIS CATs have been used in various clinical studies and integrated into electronic health records [16]. However, a practical CAT for measuring mood states in Japanese has yet to be established. Specifically, there are currently no CAT-based mood assessment tools developed based on data from persons with eating disorders or type 2 diabetes, and developing such tools may help understand their daily eating behaviors in future studies.

The present study aimed to develop a momentary CAT for real-time monitoring of mood status under daily life conditions and a CAT for mood over the most recent one-week period for use in outpatient consultations for Japanese individuals with eating disorders or type 2 diabetes.

## Methods

### Participants

The present study recruited adults (≥ 20 years old) with depression (including adjustment disorder), anxiety disorder, or an eating disorder who were attending the Department of Psychosomatic Medicine of the University of Tokyo Hospital and Nozomi Clinic, a psychiatric clinic. Adults with type 2 diabetes attending the Department of Diabetes and Metabolic Diseases of the University of Tokyo Hospital were also recruited. The recruitment was conducted between September 2014 and March 2016. We excluded the following: (1) those who were unable to understand the explanation and consent documents, (2) those who were unable to communicate, and (3) those deemed inappropriate by the physician.

Healthy individuals were also recruited through the web monitoring site, Macromill (Macromill, Inc., Tokyo, Japan) who were aged ≥ 20 years and not regularly visiting a medical institution (confirmed by self-report) in June 2014. While no other criteria were specified, only individuals who read the explanation documents and provided consent participated in the study.

### Data collection

We developed new items to assess momentary depression (74 items), anxiety (27 items), and positive affect (23 items), as well as items to assess recent one-week depression (79 items), anxiety (27 items), and positive affect (23 items). Participants were asked to rate their responses to these items on a 5-point Likert-type scale. For the momentary mood, the options were “not at all applicable,” “not very applicable,” “neutral,” “relatively applicable,” or “very applicable,” For the most recent one-week mood, the choices were “none,” “rarely,” “sometimes,” “often,” or “always.” Table 1 shows examples of these items translated from Japanese to English. In addition, participants also completed the Hospital Anxiety and Depression Scale (HADS), a widely used measurement of depression and anxiety [17].

**Table 1:** Examples of items translated from Japanese to English

Type	Item	Likert scale
Momentary depression	I feel bored.	1 = Not at all applicable; 2 = Not very applicable; 3 = Neutral; 4 = Relatiely applicable; 5 = Very applicable
Momentary anxiety	I feel anxiety.	1 = Not at all applicable; 2 = Not very applicable; 3 = Neutral; 4 = Relatiely applicable; 5 = Very applicable
Momentary positive affect	I feel satisfied.	1 = Not at all applicable; 2 = Not very applicable; 3 = Neutral; 4 = Relatiely applicable; 5 = Very applicable
Recent one-week depression	I feel bored.	1 = None; 2 = Rarely; 3 = Sometimes; 4 = Often; 5 = Always
Recent one-week anxiety	I feel anxiety.	1 = None; 2 = Rarely; 3 = Sometimes; 4 = Often; 5 = Always
Recent one-week positive affect	I feel satisfied.	1 = None; 2 = Rarely; 3 = Sometimes; 4 = Often; 5 = Always

### Overview of statistical analyses

According to analytic methods in the PROMIS project and a relevant study on CAT [18, 19], we conducted several analyses, including (1) descriptive statistics, (2) evaluation of IRT assumption, (3) fitting a graded response model (GRM) to the data, (4) evaluating differential item functioning (DIF), and (5) conducting CAT simulations. The IRT analyses excluded participants who had missing answers to any question. All analyses were performed using R version 4.3.1. Statistical significance was set at *P* < 0.05.

### Descriptive statistics

Items with unanswered categories were excluded from the analysis because their parameters could not be estimated in the IRT analysis. Additionally, items with an item-remainder correlation of < 0.3 were also excluded because they failed to meet the criteria for internal consistency.

### Evaluation of the assumptions of the IRT model

We evaluated the assumptions of the IRT model, including unidimensionality, local dependency, and monotonicity.

First, we tested unidimensionality by conducting principal component analysis (PCA). We confirmed that the proportion of variance of the first factor was ≥ 20% and that the ratio of the proportion of variance from the first to the second factor was ≥ 4. Items with low contributions to the first factor were excluded if these criteria were not met.

Next, we assessed local dependency by conducting a one-factor confirmatory factor analysis, which produced a residual correlation matrix (analyzed by the R package “lavaan,” version 0.6–16). Items with residual correlations > 0.2 were regarded as violations of local dependency. In cases of locally dependent pairs of items, we excluded the item with a lower contribution to the PCA’s first factor.

Finally, we examined monotonicity by developing a nonparametric IRT model (analyzed using the R package “mokken,” version 3.1.0). Items with scalability coefficients < 0.3 were excluded as violations of monotonicity.

### Application of the GRM

We fitted a GRM to the remaining items using the R package “mirt,” version 1.41. Discrimination and difficulty parameters for each item and latent factor θ (representing the degree of depression, anxiety, and positive affect) for each participant were estimated using maximum a posteriori. Items that had categories without maximum probability across any θ were excluded. We also examined fit statistics (S-X^2^) for each item and excluded those with a poor fit, as determined by an alpha level of 0.01.

### Evaluation of the DIF

We evaluated DIF for sex using the “DIF” function in the R package “mirt,” version 1.41.1. Items with an alpha level of < 0.01 were excluded.

### CAT simulations

After the item selection process, Cronbach’s alpha was calculated to assess internal consistency. Then, we redeveloped a GRM and recalculated discrimination and difficulty parameters for each item and the latent factor for each participant (θ_true_).

We used the resulting parameters and θ_true_ to perform a CAT simulation using the R package “catIrt” version 0.5-1. At the simulation’s onset, the estimated latent factor (θ_est_) was set to zero, and a minimum of three items were administrated.

Similar to a relevant study [19], we used Bayesian mode estimation to quantify θ, and item selection was based on unweighted Fisher information. The stopping criteria were set as follows: (a) standard error of measurement of 0.32 or (b) reaching the maximum number of items.

To assess simulation accuracy, we calculated the Pearson’s correlation coefficient (PCC) between θ_est_ and θ_true_.

### Validity of the measurements

To assess concurrent validity, we calculated PCC between θ_est_ for positive affect and HADS depression scores, θ_est_ for depression and HADS depression scores, and θ_est_ for anxiety and HADS anxiety scores.

For discriminant validity, between-group differences in the clinical and healthy groups were determined using t-tests. Specifically, comparisons were made between the healthy group and the depression group for depression scores, between the healthy group and the anxiety group for anxiety scores, and between the healthy group and the combined depression and anxiety group for positive mood scores.

## Results

### Study participants

The study included 626 participants, 312 in the clinical group (137 male, 175 female; median age 51 years) and 314 healthy individuals (158 male, 156 female; median age 47 years). Table 2 shows the participants’ characteristics. The mean (S.D.) hemoglobin A1c for participants with type 2 diabetes was 7.10 (0.80).

**Table 2:** Participant characteristics

	Number of participants	Number of female	Median age (range)
Healthy controls	314	156	47 (20–89)
Clinical group	312	175	51 (20–83)
Depression	121	67	49 (22–78)
Major depressive disorder	77	45	51 (24–78)
Adjustment disorder	44	22	43.5 (22–72)
Anxiety	65	46	45 (22–77)
Panic disorder	34	21	43.5 (22–71)
Generalized anxiety disorder	17	15	55 (26–77)
Obsessive-compulsive disorder	5	3	39 (23–46)
Social anxiety disorer	4	2	43.5 (32–65)
Others	8	7	37.5 (22–65)
Eating disorders	55	52	32 (20–58)
Anorexia nervosa restricting type	22	19	29 (21–56)
Anorexia nervosa binge-eating/purging type	20	20	29.5 (20–58)
Bulimia nervosa / Binge eating disorder	13	13	36 (20–50)
Type 2 diabetes	95	30	66 (29–83)

### CAT simulations for momentary depression

Out of the total of 626 participants, 586 completed the questionnaire without any missing values. Of the initial 74 items, 54 were ultimately selected, yielding a Cronbach’s alpha of 0.99 for these items. The CAT simulation, presented in Table 3, achieved a PCC of 0.950 (95% confidence interval [CI], 0.942–0.957) between θ_est_ and θ_true_ using an average of 4.816 items.

**Table 3:** Application of the IRT model and CAT simulation

	Number of items	Number of respondents	Average number of items administered	PCC between θ_est_ and θ_true_ (95% confidence interval)
Momentary depression	54	586	4.816	0.950 (0.942–0.957)
Momentary anxiety	6	612	3.724	0.992 (0.990–0.993)
Momentary positive affect	18	603	6.516	0.979 (0.975–0.982)
Recent one-week depression	57	584	8.038	0.969 (0.964–0.974)
Recent one-week anxiety	14	610	6.330	0.976 (0.972–0.980)
Recent one-week positive affect	16	604	4.565	0.967 (0.962–0.972)

The θ_est_ was positively correlated with the HADS depression score (Table 4). Additionally, the θ_est_ was significantly higher in the depression group than in healthy individuals (Table 5).

**Table 4:** Correlations between θ_est_ and HADS scores

	PCC between θ_est_ and HADS scores (95% confidence interval)
Momentary depression and HADS-D	0.651 (0.601–0.695)
Momentary anxiety and HADS-A	0.637 (0.587–0.682)
Momentary positive affect and HADS-D	-0.657 (-0.700–-0.609)
Recent one-week depression and HADS-D	0.689 (0.644–0.729)
Recent one-week anxiety and HADS-A	0.754 (0.718–0.787)
Recent one-week positive affect and HADS-D	-0.634 (-0.680–-0.584)

**Table 5:** Comparison of θ_est_ between the clinical groups and healthy controls for discriminant validity

Group	Mean (SD)	*P*-value
Momentary depression		
Healthy controls Depression	-0.158 (0.889) 0.397 (0.840)	< 0.001
Momentary anxiety		
Healthy controls Anxiety	-0.076 (0.784) 0.321 (0.923)	0.002
Momentary positive affect		
Healthy controls Depression and Anxiety	0.119 (0.895) -0.279 (0.934)	< 0.001
Recent one-week depression		
Healthy controls Depression	-0.218 (0.955) 0.667 (0.800)	< 0.001
Recent one-week anxiety		
Healthy controls Anxiety	-0.153 (0.805) 0.485 (0.766)	< 0.001
Recent one-week positive affect		
Healthy controls Depression and Anxiety	0.072 (0.950) -0.185 (0.863)	0.003

### CAT simulations for momentary anxiety

Out of the participants, 612 completed the questionnaire without missing values. From the initial 27 items, six were ultimately selected, resulting in a Cronbach’s alpha of 0.94. The results of the CAT simulation are presented in Table 3, achieving a PCC of 0.992 (95% CI, 0.990–0.993) between θ_est_ and θ_true_ using an average of 3.724 items.

The θ_est_ was positively correlated with the HADS anxiety score (Table 4). Furthermore, the θ_est_ was significantly higher in the anxiety group than in healthy individuals (Table 5).

### CAT simulations for momentary positive affect

Out of all participants, 603 completed the questionnaire without missing values. Among the initial 23 items, 18 were selected, resulting in a Cronbach’s alpha of 0.93. The CAT simulation, shown in Table 3, achieved a PCC of 0.979 (95% CI, 0.975–0.982) between θ_est_ and θ_true_ using an average of 6.516 items.

The θ_est_ was negatively correlated with the HADS depression score (Table 4). The θ_est_ was significantly lower in the depression and anxiety group than in healthy individuals (Table 5).

### CAT simulations for most recent one-week depression

Out of all participants, 584 completed the questionnaire without missing values. From the initial 79 items, 57 items were selected, with a Cronbach’s alpha of 0.99. The CAT simulation, shown in Table 3, achieved a PCC of 0.969 (95% CI, 0.964–0.974) between θ_est_ and θ_true_ with an average of 8.038 items.

The θ_est_ was positively correlated with the HADS depression score (Table 4). In addition, the θ_est_ was significantly higher in the depression group than in healthy participants (Table 5).

### CAT simulations for most recent one-week anxiety

Out of all participants, 610 completed the questionnaire without missing values. From the original 26 items, 14 were selected, resulting in a Cronbach’s alpha of 0.97. The CAT simulation, as detailed in Table 3, achieved a PCC of 0.976 (95% CI, 0.972–0.980) between θ_est_ and θ_true_ using an average of 6.330 items.

The θ_est_ was positively correlated with the HADS anxiety score (Table 4). Furthermore, the θ_est_ was significantly higher in the anxiety group than in healthy participants (Table 5).

### CAT simulations for most recent one-week positive affect

Out of all participants, 604 completed the questionnaire without missing values. Among the 23 items, 16 were ultimately selected, yielding a Cronbach’s alpha of 0.95. The CAT simulation achieved a PCC of 0.967 (95% CI, 0.962–0.972) between θ_est_ and θ_true_ using an average of 4.565 items (Table 3).

The θ_est_ was negatively correlated with the HADS depression score (Table 4). Additionally, the θ_est_ was lower in the depression and anxiety group than in healthy individuals (Table 5).

## Discussion

In the present study, we prepared six types of item pools for the CAT based on IRT. After item selection to satisfy the assumptions of IRT, the items exhibited a high Cronbach’s alpha coefficient, indicating adequate internal consistency. The CAT simulations demonstrated a strong correlation between estimated latent factors based on a small number of items and those based on the full item set. Each scale had sufficient concurrent validity with the HADS scores and discriminant validity between the clinical group and healthy controls.

The CAT developed in this study has the potential to improve measurement accuracy and reduce the time needed for assessment. Additionally, automated scoring can reduce the burden on clinicians and provide immediate feedback to testees. It would be desirable to apply the CAT to monitor changes in psychological status and provide prompt feedback. Notably, the momentary version is promising for detecting psychological status in daily life, which has been a black box in clinical practice. This approach can also evaluate longitudinal relations between psychological status and eating behaviors, potentially enabling clinicians to offer more tailored treatment.

Individuals with depression and anxiety disorders exhibited higher scores for depression and anxiety and lower scores for positive affect than healthy individuals, both in the momentary and most recent one-week, confirming the scales’ discriminant validity.

The present study has several limitations. First, while the minimum sample size for the GRM is set at 500 cases [20], which this study met, a larger sample size would be preferable. This study did not fully examine the severity of each disease. Incorporating more severe cases may allow more accurate measurement across a broader spectrum of characteristics. Second, the questionnaire was designed to take about 20 min to complete; however, some participants left incomplete questions, possibly due to boredom or a desire to finish quickly, which may have influenced the results. Third, although the momentary version of the CAT aimed to assess psychological status in daily life, the present study conducted a one-time survey without specifying any particular situation. The absence of context regarding the circumstances under which participants responded could have influenced the outcomes. Fourth, this study does not include a detailed assessment of participants’ eating behaviors or daily activities. Future studies should investigate the association between daily mood status and eating behaviors using the developed CAT. Fifth, we did not evaluate the presence of mental disorders in persons with type 2 diabetes. Finally, we did not evaluate the treatment stage or duration of illness for participants with eating disorders.

In conclusion, we successfully developed a momentary version of CAT for real-time monitoring of mood status in daily life and a CAT for assessing the most recent one-week mood status that is suitable for outpatient consultations for individuals with eating disorders or type 2 diabetes.

## Electronic supplementary material

Below is the link to the electronic supplementary material.

Supplementary Material 1

## Declarations

### Data sharing

The datasets analyzed during the current study are not publicly available because the approval of data sharing has not been obtained from the institutional review board but are available from the corresponding authors on reasonable request.

## Abbreviations

CAT: Computerized adaptive testing
DIF: Differential item functioning
GRM: Graded response model
HADS: Hospital Anxiety and Depression Scale
IRT: Item response theory
PCA: Principal component analysis
PCC: Pearson’s correlation coefficient
PROMIS: Patient-Reported Outcomes Measurement Information System
