# Supplementary material for: Development of computer adaptive tests to assess the psychological status of individuals with an eating disorder or type 2 diabetes
Source: Biopsychosoc Med. 2025 Feb 14;19:2. doi: 10.1186/s13030-025-00325-z (PMC11827128; doi:10.1186/s13030-025-00325-z)
Supplement: Supplementary file 1 — Supplementary Material 1 [file 13030_2025_325_MOESM1_ESM.pdf]

The University of Tokyo

Department of Psychosomatic Medicine
The University of Tokyo Hospital

7-3-1 Hongo, Bunkyo-ku
Tokyo, JAPAN 113-8655

January 31, 2025

Editor-in-chiefs

BioPsychoSocial Medicine

Dear Editor-in-chiefs:

I wish to re-submit our manuscript titled "**Development of computer adaptive tests to assess the psychological status of individuals with an eating disorder or type 2 diabetes**." The manuscript ID is BPSM-D-24-00076R3.

We sincerely appreciate you and the reviewers for reviewing our manuscript and the thoughtful suggestions. We have revised the manuscript and made all the necessary changes in accordance with the suggestions of Reviewer #3. These changes are highlighted in red in the revised manuscript.

Thank you for your consideration. I look forward to hearing from you.

Sincerely,

Kazuhiro Yoshiuchi, MD, PhD
Department of Stress Sciences and Psychosomatic Medicine,
Graduate School of Medicine,
The University of Tokyo, 7-3-1, Hongo, Bunkyo-ku, Tokyo 113-8655 Japan
Phone: +81-3-5800-9764
Email: kyoshiuc-tky@umin.ac.jp
